# Proteomic Profiling of Mouse Brain Pyruvate Kinase Binding Proteins: A Hint for Moonlighting Functions of PKM1?

**DOI:** 10.3390/ijms24087634

**Published:** 2023-04-21

**Authors:** Olga Buneeva, Arthur Kopylov, Oksana Gnedenko, Marina Medvedeva, Alexander Veselovsky, Alexis Ivanov, Victor Zgoda, Alexei Medvedev

**Affiliations:** 1 Institute of Biomedical Chemistry, 10 Pogodinskaya Street, Moscow 119121, Russia; 2 Department of Biochemistry, School of Biology, Moscow State University, Moscow 119991, Russia

**Keywords:** pyruvate kinase, pyruvate kinase isoforms, PKM, moonlighting functions, PKM binding proteins, interactome

## Abstract

Affinity-based proteomic profiling is widely used for the identification of proteins involved in the formation of various interactomes. Since protein–protein interactions (PPIs) reflect the role of particular proteins in the cell, identification of interaction partners for a protein of interest can reveal its function. The latter is especially important for the characterization of multifunctional proteins, which can play different roles in the cell. Pyruvate kinase (PK), a classical glycolytic enzyme catalyzing the last step of glycolysis, exists in four isoforms: PKM1, PKM2, PKL, and PKR. The enzyme isoform expressed in actively dividing cells, PKM2, exhibits many moonlighting (noncanonical) functions. In contrast to PKM2, *PKM1*, predominantly expressed in adult differentiated tissues, lacks well-documented moonlighting functions. However, certain evidence exists that it can also perform some functions unrelated to glycolysis. In order to evaluate protein partners, bound to PKM1, in this study we have combined affinity-based separation of mouse brain proteins with mass spectrometry identification. The highly purified PKM1 and a 32-mer synthetic peptide (PK peptide), sharing high sequence homology with the interface contact region of all PK isoforms, were used as the affinity ligands. This proteomic profiling resulted in the identification of specific and common proteins bound to both affinity ligands. Quantitative affinity binding to the affinity ligands of selected identified proteins was validated using a surface plasmon resonance (SPR) biosensor. Bioinformatic analysis has shown that the identified proteins, bound to both full-length PKM1 and the PK peptide, form a protein network (interactome). Some of these interactions are relevant for the moonlighting functions of PKM1. The proteomic dataset is available via ProteomeXchange with the identifier PXD041321.

## 1. Introduction

Affinity-based proteomic profiling is a powerful approach used in proteomic studies for separation of various groups of proteins [1]. The combination of affinity chromatography with mass spectrometry represents an effective tool for the generation of a broad range of data for protein–protein interactions (PPIs), mapping post-translational modifications or recovering a certain target from the whole proteome, characterization of intracellular signaling networks and particular interactomes, etc. [2,3,4,5,6]. 

In the context of PPIs, so-called moonlighting proteins attract much interest because these proteins exhibit more than one function in the cell [7]. The repertoire of protein partners interacting with such moonlighting proteins significantly differs in the dependence of particular biochemical/physiological processes in which the moonlighting protein is involved. Currently, several hundred moonlighting proteins have been recognized [7,8,9,10]. 

Glycolytic enzymes are one of the most abundant groups of moonlighting proteins [10,11]. Pyruvate kinase (PK, EC 2.7.1.40) is a highly conserved enzyme catalyzing the irreversible conversion of ADP and phosphoenolpyruvate to ATP and pyruvate (substrate-level phosphorylation reaction). Mammalian PK exists in four isoforms differed by expression patterns and regulatory properties: PKM1, PKM2, PKL, and PKR [12,13]. PK is most active as a tetramer, and dissociation into dimers and monomers results in its inactivation [13]. The isoform PKM2 is typical of embryonic and adult dividing cells [14]. Besides its role in glycolysis, PKM2 performs many moonlighting functions and participates in different cellular processes, such as: transcription, translation, signaling, and cytoskeletal dynamics [10,13,14,15,16,17,18,19,20]. In contrast to PKM2, PKM1 lacks well-documented moonlighting functions [14]. It is predominantly expressed in adult differentiated tissues including brain and musculature [12], and skeletal muscles are frequently used as the source for purification of this enzyme [21,22]. In the mouse brain, PKM1 was found in gray matter neurons and white matter bundles [23], but no functions of PKM1 unrelated to glycolysis have been reported so far. 

Certain evidence exists that PKM1 can perform some functions unrelated to its role in glycolysis. For example, a PKM1 monomer is known as a thyroid hormone (T3) binding protein [24]. PKM1 can bind to ribosomes [20,25]. In a rabbit reticulocyte in vitro translation system, exogenously added PKM1-GST decreased the translation of some mRNAs [25]. In addition, PKM1 can form hetero-oligomers with other PK isoforms [13]. This suggests an important role of oligomer-to-monomer transition and the interface contact region that could be potentially involved in PKM1 functioning unrelated to glycolysis. 

Since classical and moonlighting functions obviously require different sets of protein partners, proteomic profiling with the protein of interest as an affinity ligand may give a hint for further search and research of its moonlighting. The latter is especially interesting in the context of PKM1, which shares high sequence similarity with PKM2 [26] and therefore could be considered “as a substitute player in the PKM2 game”. Thus, the aim of this study was to investigate profiles of mouse brain proteins bound to the highly purified PKM1 or a synthetic 32-mer linear PK peptide. This PK peptide corresponds to the interface of PKL/R isoforms and exhibits high homology with PKM isoforms. Results of this study suggest that the profiles of proteins bound to either purified PKM1 or the PK peptide significantly differ. However, common proteins, bound to both full-length PKM1 and the PK peptide, form a protein network (interactome) that may reflect some moonlighting functions of PKM1. 

## 2. Results

### 2.1. Proteomic Profiling of Mouse Brain Proteins Using PKM1 as the Affinity Ligand 

Proteomic profiling of cleared lysates of mouse brain homogenates performed using immobilized PKM1 as the affinity ligand resulted in confident identification of 44 individual proteins including brain PKM (Table 1, Supplementary Materials Table S1). This suggests that the immobilized affinity ligand, purified rabbit muscle PKM1, was functionally competent to form PKM oligomers. All the identified brain proteins belonged to the following functional groups: (1) metabolic enzymes; (2) proteins involved in cytoskeleton formation and trafficking; (3) proteins involved in signal transduction and enzyme activity regulation; (4) protective proteins and components of the ubiquitin–proteasome system; and (5) protein regulators of gene expression, cell division, and differentiation.

### 2.2. Biosensor Validation of PKM1 Interaction with Selected Identified PKM Binding Proteins 

**Figure 1:**
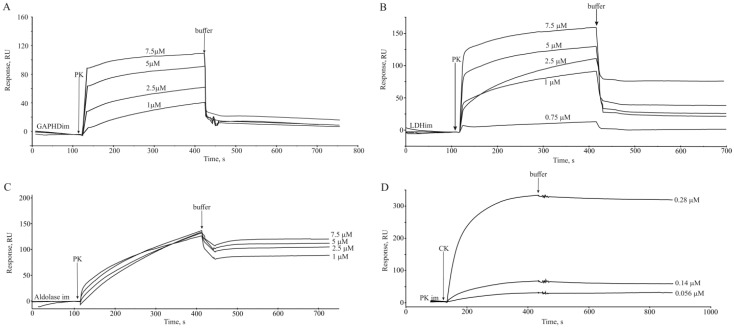
Biosensor validation of PKM1 interaction with selected identified PKM binding proteins. In the case of A, B, and C, purified rabbit muscle PK1 was used as an analyte, and purified rabbit muscle GAPDH (**A**), LDH (**B**), and aldolase A (**C**) were immobilized onto a chip surface; in (**D**) PKM1 was immobilized on the chip surface and CK was used as the analyte.

Interaction of some of the identified PKM binding proteins with PKM1 has been validated in the optical biosensor experiments using available purified rabbit muscle enzymes: PKM1, aldolase, glyceraldehyde 3-phosphate dehydrogenase (GAPDH), lactate dehydrogenase (LDH), and creatine phosphokinase (CK). Correctness of extrapolation of the results obtained using the rabbit muscle enzyme for validation of PKM1 interaction is determined by high similarity between mouse and rabbit glycolytic enzymes [27]. All the selected proteins demonstrated quantitative binding to the PKM1 (Figure 1), with the K_d_ values ranging from 10^−^^8^ M (CK) to about 5·10^−^^6^ M (LDH) (Table 2). 

### 2.3. Proteomic Profiling of Mouse Brain Proteins Using the PK Peptide as the Affinity Ligand 

Identification of PKM as the protein bound to the immobilized PKM1 (PKM1-binding protein) suggested involvement of a PKM oligomer-forming interface in this interaction. In order to investigate the role of the PK interface in the PKM proteome formation we have compared profiles of mouse brain proteins bound to PKM1 and the PK peptide. The PK peptide corresponds to the sequence fragment (residues 406–437) of PKR/L and shares high homology with PKM1 and PKM2.

Proteomic profiling of cleared lysates of mouse brain homogenates performed using the immobilized PK peptide as the affinity ligand resulted in confident identification of more than 75 individual proteins (Supplementary Table S2). These proteins fell into the same functional groups as the mouse proteins bound to the full-length PKM1. Although the total number of brain proteins bound to the PK peptide was significantly higher than in the case of PKM1, 15 proteins were common for both affinity ligands (Table 3). At least 10 of 15 common proteins are known to exhibit some moonlighting functions (Table 3). 

**Figure 2:**
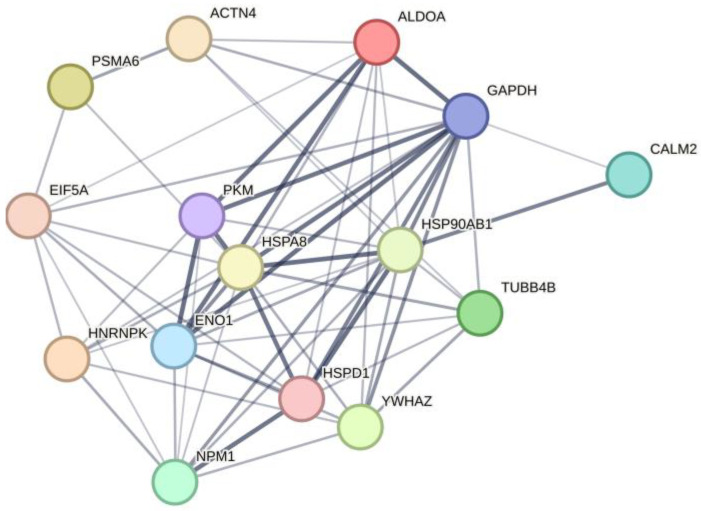
The interactome map reflecting the interaction between mouse brain proteins bound to the full-length PKM1 and the PK peptide. Explanations are given in the text.

The commonly identified proteins bound to both the PK peptide and full-length PKM1 were submitted for plotting an interactome map. The resultant map contained 15 nodes and 60 edges in the interactome (Figure 2). The core of the interactions was formed by glycolytic proteins exhibiting moonlighting functions (ALDOA, GAPDH, ENO1, and PKM) and a group of proteins involved in protein folding and chaperoning (HSP90AB1, HSPA8, and NPM1). Results of our study indicate direct interactions between PKM1 and the mouse brain proteins with documented moonlighting functions. These proteins may be relevant for the performance of moonlighting functions of PKM1. 

## 3. Discussion

Performance of canonical and moonlighting functions by multifunctional proteins obviously involves different sets of proteins and PPIs. In the case of unknown moonlighting functions of a protein of interest, certain information could be obtained by analyzing its protein partners and PPIs. 

In the context of putative moonlighting functions, PKM1, an enzyme of adult non-proliferating tissues (skeletal and cardiac musculature, and the brain), is always “in the shadow” of PKM2, expressed by embryonic and adult actively proliferating tissues. Being encoded by a single gene (*PKM*), PKM1 and PKM2 are formed due to alternative splicing: the alternative exons encode peptide products of the same length, but their identity is about 50% [28]. Moonlighting functions of PKM2 are associated with various cellular processes. Mitogenic and oncogenic stimulation of proliferating cells induces PKM2 translocation into the nucleus [29,30], where it functions as a transcriptional coactivator and a protein kinase phosphorylating histone [31,32]. 

The moonlighting role of PKM1 is poorly investigated and there are a few examples suggesting that PKM1 could have moonlighting functions in some cells. In this case proteomic profiling with PKM1 as the affinity ligand may help to find putative protein partners for such moonlighting functions. 

Using highly purified skeletal rabbit PKM1 and the synthetic 32-mer PK peptide as the affinity ligands, we have identified 44 and 92 individual mouse brain proteins, respectively (Table 1 and Table 3, and Supplementary Tables S1 and S2). PKM has been identified among common mouse brain proteins bound to both affinity sorbents (PKM1 and the PK peptide, Table 3). This suggests functional importance of the interface contact region for interaction of PKM1 with potential protein partners. The higher repertoire of proteins bound to the PK peptide may be explained by the higher conformational accessibility of the peptide interaction sites. Nevertheless, there were common proteins bound to both affinity ligands and formed several groups of functional interactions (Figure 2). 

PKM1 participates in interactions with proteasome subunits (PSMA6), known glycolytic proteins exhibiting moonlighting functions (ALDOA, GAPDH, and ENO1), and also with proteins involved in protein folding and chaperoning (HSP90AB1, HSPA8, and NPM1). Some of these interactions obviously reflect canonical functions of the glycolytic enzymes supporting proteasomes with additional energy resources and promoting maintenance of nucleotide-dependent functions of proteasomes [33]. The group of proteins involved in protein folding and chaperoning is particularly interesting. It includes several chaperones, HSP90AB1, HSPA8, HSPD1, and NPM1, directly interacting with PKM1. Nucleophosmin (NPM1) is a multifunctional protein, which shuttles between nucleoli, nucleoplasm, and cytoplasm and performs its multifaceted roles (see for review [34]). It exhibits histone- and protein-chaperone activity, participates in DNA replication and repair, ribosome assembly and export, and centrosome duplication and cell cycle control [34,35,36,37,38,39,40,41]. In nucleoli NPM1 acts as a hub protein, which contributes to nucleolar organization through multiple (heterotypic and homotypic) interactions [34]. The number of NPM1-binding proteins exceeds several dozens, and their list is constantly growing [34]. It is possible that PKM1 interaction with NPM1 and other proteins has a regulatory importance in various cell compartments including the nucleus. 

Convincing evidence exists in the literature that PKM2, but not PKM1, can move into the nucleus due to the exclusive presence of exon 10, encoding the nuclear localization signal [31,32]. Since the nuclear localization signal is located in the interface contact region, it appears that realization of this scenario requires dissociation of PKM2 tetramers. However, recently it has been demonstrated that under certain conditions PKM1 can also be translocated to the nucleus [42] and therefore interact with potential nuclear targets. 

Thus, results of this proteomic profiling have shown that, in addition to proteins related to the classical functioning of PKM1, there are other PKM1-binding proteins. These proteins may be relevant to some activities unrelated to the role of PKM1 as an important glycolytic enzyme. Since binding capacities obviously involve the PKM1 interface contact region, this suggests an important role of oligomer-to-monomer transition for PKM1 moonlighting.

## 4. Materials and Methods

### 4.1. Reagents

CNBr-activated Sepharose 4B, creatine kinase from rabbit muscle, dithiothreitol, iodoacetamide, Tris (hydroxymethyl) aminomethane, urea, guanidine hydrochloride, glycerol, a cocktail of protease inhibitors, 4-vinylpyridine, sodium deoxycholate, ammonium bicarbonate, sodium chloride, Triton X-100, and Coomassie brilliant Blue were from Merck (Branchburg, NJ, USA); acetonitrile was from Fisher Chemical (Loughborough, Leicestershire, UK); sodium acetate, boric acid, formic acid, sodium tetraborate, and sodium hydroxide were from Acros Organics (Morris Planes, NJ, USA); modified trypsin (sequencing grade) was purchased from Promega (Madison, WI, USA); 10 kDa membrane filters were from Sartorius Stedium Biotech (Goettingen, Germany); Amicon Ultracel-10K centrifugal concentration filters were from Millipore (Burlington, MA, USA); and Acclaim PepMap^®^ RSLC C18 column (150 mm × 75 μm, particle size 2 μm, pore size 100 Å) were from Dionex (Sunnyvale, CA, USA). The remaining reagents of the highest degree of purity were obtained from local suppliers. 

Reagents for the Biacore biosensor were obtained from Cytiva (Marlborough, MA, USA). These included HBS-EP buffer (150 mM NaCl, 3 mM EDTA, 0.005% surfactant P20, 10 mM HEPES, pH 7.4); 10 mM acetate buffer, pH 4.0, pH 5.0, and pH 5.5; and amine coupling reagents kit, containing 1–ethyl–3–(3–dimethylaminopropyl) carbodiimide hydrochloride (EDC), N–hydroxysuccinimide (NHS), and 1 M ethanolamine–HCl, pH 8.5.

Electrophoretically homogeneous PKM1, GAPDH, aldolase A, and LDH were isolated from skeletal muscles of adult (five months old) rabbits according to [22]. The specific activity of the enzyme preparations was 295 μmol/min per mg of protein (PKM1), 170 μmol/min per 1 mg of protein (evaluated in the reaction of 3-phosphoglycerate reduction by NADH; (GAPDH), 10.4 μmol/min per 1 mg of protein (aldolase A), 28 μmol/min per 1 mg of protein (LDH). Before use, the purified enzymes were kept as an ammonium sulfate suspension at 4 °C for not more than two months. 

### 4.2. Selection of the PK Peptide 

The PK peptide synthesized by Immunotex (Stavropol, Russia; custom made order) corresponds to the sequence fragment (residues 406–437) of PKR/L. It shares high homology with PKM1 and PKM2 (See Supplementary Materials Figure S1). 

### 4.3. Animals

Adult (three months old) male C57BL/6 mice (weighing 20–25 g) obtained from the Stolbovaya nursery (Moscow region), were used in this study. Experiments were performed one week after their arrival from the nursery. Animals were maintained at natural illumination and had free access to standard laboratory chow and water. All procedures conform to the Russian version of the Guide for the Care and Use of Laboratory Animals (Washington, DC, USA, 1996) and have been approved by the Animal Care and Use Committee at the Institute of Biomedical Chemistry.

### 4.4. Preparation of Brain Homogenate Lysates

After decapitation of mice under light ether anesthesia, the brain tissue was homogenized using a SilentCrusher S homogenizer (Heidolph, Wood Dale, IL, USA) at 50,000 rpm in 0.05 M potassium phosphate buffer, pH 7.4 (buffer A), to obtain a 30% homogenate. After addition of Triton X-100 (final concentration 3%), incubation for 60 min at 4 °C, and subsequent three-fold dilution with buffer A, the samples were centrifuged at 16,000× *g* for 30 min. The resultant supernatant (lysate) was used for affinity chromatography on Sepharose with the immobilized PKM1 for subsequent mass spectrometric analysis.

### 4.5. Immobilization of PKM1 and the PK Peptide on Cyanogen Bromide-Activated Sepharose 4B, Affinity Chromatography and Sample Preparation for Mass Spectrometric Analysis

Immobilization of PKM1 and the PK peptide onto Cyanogen Bromide-Activated Sepharose 4B (CNBr-Sepharose) was carried out according to the previously described protocol [43]. For determination of proteins nonspecifically bound to the sorbent, a control CNBr-Sepharose was used. It was subjected to the same procedures but without the addition of PKM1.

Lysates of brain homogenates (protein concentration of 6 mg/mL) were added to the affinity resin washed with buffer A. The suspension (1:1), containing a cocktail of protease inhibitors added at a concentration recommended by the manufacturer, was incubated overnight at 4 °C and gentle stirring. The affinity resin was then washed with 100 volumes of buffer A to remove nonspecifically bound proteins (protein content in the washings was controlled by OD_280_). The remaining proteins were eluted at room temperature with 0.1 M glycine buffer, pH 2.8, containing 3 M NaCl (at a flow rate of 0.5 mL/min), using a column 1 cm × 2 cm. The eluate (30 mL) was concentrated to 0.25 mL using an Amicon Ultra centrifuge device. Proteins were extracted with a mixture of chloroform–methanol [44]. The reduction of disulfide bonds, the alkylation of sulfhydryl groups, and trypsinolysis were performed on Vivaspin 500 centrifuge filters with a 10,000 Da membrane, as described in [44]. Samples were evaporated using a 5301 vacuum concentrator (Eppendorf, Hamburg, Germany), dissolved in 0.1% formic acid and analyzed using liquid chromatography tandem mass spectrometry (LC-MS/MS). 

### 4.6. The Mass Spectrometric Analysis (LC-MS/MS)

The mass spectrometric analysis was performed using an Ultimate 3000 RSLCnano (Thermo Scientific, Waltham, MA, USA) integrated system for high-performance liquid separation of peptides in the nanoflow mode. Chromatographic separation of peptides was carried out on an analytical reverse phase column Acclaim Pepmap^®^ C18 (75 µm × 150 mm, 2 µm particle size, Thermo Scientific, USA) in a linear elution gradient of mobile phase A (0.1% aqueous solution of formic acid) and mobile phase B (80% acetonitrile, 0.1% formic acid) from 2% to 40% at a flow rate of 0.3 μL/min for 60 min, followed by equilibration of the chromatographic system in the initial conditions of the gradient (A:B = 2:98) for 5 min.

A Thermo Scientific Q Exactive HF-X mass spectrometer equipped with a nanoelectrospray ionization source (nESI) was operated in the positive ionization mode with a resolution of 120,000 at *m*/*z* 200, the ion accumulation volume in the trap was set to 1 × 10^6^, the ion accumulation time in the trap was maximum 50 ms. The dominant charge state of precursor ions was set as 2^+^, charge states above 4^+^ and below 2^+^ were excluded from further analysis. Scanning of tandem spectra was carried out in the mode of automatic selection of 20 dominant peaks of precursor ions recorded at *m/z* = 350–1400. The resolution for detecting fragment ions was set to 15,000 at *m/z* 200, the ion accumulation volume in the trap was 1 × 10^5^, and the ion accumulation time in the trap was a maximum of 50 ms. Parent ions were isolated in a window of 2.0 *m/z* offset by 0.5 *m/z* for better isotope capture. Measured precursor ions were excluded from subsequent analysis for 20 s after scanning. The obtained mass spectrometric data with the *.raw extension were processed by the MaxQuant software (v 1.6.3.4) with the built-in Andromeda search algorithm. Mouse (*Mus musculus*) Swiss Prot/Uniprot complete proteome protein sequence database was downloaded from the Uniprot database with the addition of reversed sequences and commonly encountered contaminating sequences to apply the target decoy approach. The method was used to calculate the FDR (False Discovery Rate) parameter; the FDR parameter of 1% was taken as a cutoff for protein registration. The following parameters were used for signal extraction and its subsequent processing: the proteolytic cleavage enzyme was trypsin; the maximum allowable amount of intrapeptide residues of lysine or arginine was not more than 1; and the allowable error in measuring the monoisotopic mass of the peptide was ±0.01 Da, and the allowable error in measuring the fragment ion was ±0.05 Da. Carbamide methylation of cysteine residues was chosen as a fixed chemical modification, and methionine oxidation was chosen as a variable modification [20]. 

The relative abundance (quantitation) of the proteins defined as an emPAI value (Exponentially Modified Protein Abundance index) was based on the protein coverage by the peptide matches in a database search result. The obtained proteome was percolated to extract proteins for bioinformatic analysis. The confidence score for these proteins was at least 95%.

Each protein presented in the tables was identified in at least three independent experiments with a Bonferroni-adjusted *p*-value cut-off of 0.001 (raw *p*-value cut-off 0.01). Protein function identification was performed using UniProt functional information. 

### 4.7. Biosensor Analysis 

#### 4.7.1. Immobilization of Proteins

Measurements were performed with a Biacore T200 instrument (Cytiva, Marlborough, MA, USA), thermostated at 25 °C. The surface of the CM5 chip was activated by a mixture 0.2 M 1-ethyl-3(3-dimethylaminopropyl)-carbodiimide hydrochloride/0.05 M N-hydroxysuccinimide for 7 min at 5 µL/min. The 50 µg/mL samples of pyruvate kinase, LDH, aldolase, or GAPD in 10 mM acetate buffer, pH 5.0, pH 4.0, or pH 5.5, respectively, were injected over an activated chip surface for 10 min at 5 µL/min followed by a 2 min injection of HBS-EP buffer to remove excess ligand, and a 2 min injection of 1 M ethanolamine, pH 8.5, to inactivate residual active groups.

#### 4.7.2. Binding Measurements

Protein–protein interactions were monitored by injecting proteins dissolved at various concentrations in buffer A (running buffer) at the flow rate of 10 µL/min for 5 min. The sensor surface was regenerated between sample injections by washing with 1 M NaCl in running buffer for 0.5 min at the flow rate of 50 µL/min. Interactions were estimated by subtracting the response in a blank flow cell from the response in a cell with immobilized ligands.

Data analysis was performed using the BIAevaluation v.4.1 software. Kinetic rate constants were calculated from the sensorgrams by globally fitting response curves obtained at various ligand concentrations to the 1:1 binding model.

### 4.8. Bioinformatic Analysis of Protein–Protein Interactions 

Analysis of protein interactions was performed using the STRING (version 11.5) tool. Common proteins interacting with both full-length PKM1 and the PK peptide were submitted for plotting an interactome map with at least 0.7 confidence interaction score. Pathways and processes were picked up from the KEGG and Reactome databases provided that FDR was <1.0 × 10^−5^, the interaction strength coefficient was more than 1.0, and a subset of at least four different members of the network fell into the certain pathway or process. We selected the most preferable signaling pathways and biological processes that fit the proposed network of interactions. The resultant map contained 15 nodes and 60 edges in the interactome (with the confidence level at least 0.40 and higher, and the average local clustering coefficient of 0.698). 

## 5. Conclusions

The affinity-based proteomic profiling of mouse brain proteins, performed by using highly purified PKM1 as the affinity ligand, resulted in the identification of 44 PKM1-binding proteins. Since one of the identified proteins was PKM, the immobilized affinity ligand, purified rabbit muscle PKM1, was functionally competent to form PKM oligomers. This suggests involvement of the interface contact region in the interaction with PKM binding proteins. The use of the 32-mer peptide (PK peptide) significantly increased the repertoire of brain proteins bound to this affinity ligand. Nevertheless, there were 15 common proteins bound to both PKM1 and the PK peptide. These common proteins form a protein network (interactome) and some interactions especially with proteins involved in protein folding and chaperoning are relevant for moonlighting functions of PKM1.

## Figures and Tables

**Table 1:** Distribution of the identified mouse brain proteins among functional groups.

Group No.	Protein Functions	Number of Identified Proteins Bound to	
		Immobilized Full-Length PKM1	Immobilized PK Peptide
1	metabolic enzymes	13	8
2	proteins involved in cytoskeleton formation and trafficking	10	29
3	proteins involved in signal transduction and enzyme activity regulation	6	19
4	protective proteins and components of the ubiquitin–proteasome system	6	14
5	protein regulators of gene expression, cell division, and differentiation	9	22
total		44	92

**Table 2:** Biosensor validation of the interaction of selected proteins with pyruvate kinase.

Immobilized Ligand	Analyte	ka, M^−1^·s^−1^	kd, s^−1^	K_d_, M
Pyruvate kinase	Creatine kinase B-type	(8.64 ± 0.03) × 10^4^	(9.24 ± 0.18) × 10^−4^	1.07 × 10^−8^
Glyceraldehyde-3-phosphate dehydrogenase	Pyruvate kinase	(1.53 ± 0.17) × 10^3^	(1.62 ± 0.05) × 10^−3^	1.06 × 10^−6^
Lactate dehydrogenase	Pyruvate kinase	(1.37 ± 0.08) × 10^3^	(6.57 ± 0.13) × 10^−3^	4.8 × 10^−6^
Aldolase A	Pyruvate kinase	(1.69 ± 0.06) × 10^3^	(2.38 ± 0.1) × 10^−3^	1.41 × 10^−6^

**Table 3:** Common mouse brain proteins interacting with the full-length PKM1 and the synthetic PK peptide *.

no.	Accession Number	Gene	Protein Name (Uniprot)	Functional Group
**1**	Q61937	*NPM*	Nucleophosmin	5
**2**	**P52480**	***KPYM***	**Pyruvate kinase PKM**	1
**3**	**P61979**	***HNRPK***	**Heterogeneous nuclear ribonucleoprotein K**	5
**4**	**P63017**	***HSP7C***	**Heat shock cognate 71 kDa protein**	4
**5**	**P63101**	***1433Z***	**14-3-3 protein zeta/delta**	3
**6**	**P63038**	***CH60***	**60 kDa heat shock protein, mitochondrial**	4
**7**	**P0DP26**	***CALM1***	**Calmodulin-1**	3
**8**	**P16858**	***G3P***	**Glyceraldehyde-3-phosphate dehydrogenase**	1
**9**	**P17182**	***ENOA***	**Alpha-enolase**	1
**10**	P63242	*IF5A1*	Eukaryotic translation initiation factor 5A-1	5
**11**	P57780	*ACTN4*	Alpha-actinin-4	2
**12**	Q9QUM9	*PSA6*	Proteasome subunit alpha type-6	4
**13**	**P11499**	***HS90B***	**Heat shock protein HSP 90-beta**	4
**14**	**P05064**	***ALDOA***	**Fructose-bisphosphate aldolase A**	1
**15**	P68372	*TBB4B*	Tubulin beta-4B chain	2

* Moonlighting proteins are shown in bold. Numbers in the functional group column designate the following protein functions: 1. metabolic enzymes; 2. proteins involved in cytoskeleton formation and trafficking; 3. proteins involved in signal transduction and enzyme activity regulation; 4. protective proteins and components of the ubiquitin–proteasome system; 5. protein regulators of gene expression, cell division, and differentiation.

## Data Availability

The mass spectrometry proteomics data have been deposited in the ProteomeXchange Consortium via the PRIDE partner repository with the dataset identifier PXD041321.

## Supplementary Materials

The following supporting information can be downloaded at: https://www.mdpi.com/article/10.3390/ijms24087634/s1.
