# Supplementary material for: Proteomic Profiling of Mouse Brain Pyruvate Kinase Binding Proteins: A Hint for Moonlighting Functions of PKM1?
Source: Int J Mol Sci. 2023 Apr 21;24(8):7634. doi: 10.3390/ijms24087634 (PMC10143413; doi:10.3390/ijms24087634)
Supplement: Supplementary file 1 [file ijms-24-07634-s001.zip › ijms-2325377-supplementary.pdf]

SUPPLEMENTARY FIGURE

PKM1 394 LFEELVRASSHSTDLMEAMAMGSVEASYKCLA 425

PKM2 394 LFEELRRLAPITSDPTEATAVGAVEASFKCCS 425

PKR/L 406 LFEELRRAAPLSRDPTEVTAIGAVEAAFKCCA 437

Figure S1. Alignment of amino acid sequences of PKM1, PKM2, and PKR/L. The synthetic PK peptide used in this study corresponds to the sequence of PKR/L.

SUPPLEMENTARY TABLES S1 and S2

Supplementary Table S1. Individual mouse brain proteins exclusively bound to PKM1 immobilized on CNBr-Sepharose

#	Accession number	Gene	Recommended protein name (UniProt)	Peptides	Unique peptides	Sequence coverage, %	Confidence, % *	emPAI †	Adjusted p-value‡	Protein function
1	O35490	*BHMT*	Betaine--homocysteine S-methyltransferase 1	2	2	8.6	100	25	2.55E-04	1
2	P50396	*GDI1*	Rab GDP dissociation inhibitor alpha	2	2	4.47	100	26.6	1.59E-04	3
**3**	P11798	*CAMK2A*	Calcium/calmodulin-dependent protein kinase type II subunit alpha	1	1	6.28	100	46.5	2.54E-04	3
**4**	**P16125**	*LDHB*	**L-lactate dehydrogenase B chain**	2	2	10.48	100	22.1	1.53E-04	1
5	P05214	*TUBA3A*	Tubulin alpha-3 chain	7	0	22.89	99.77	18.6	1.56E-04	2
6	P40124	*CAP1*	Adenylyl cyclase-associated protein 1	1	1	3.8	100	31.9	1.71E-04	2
**7**	**P07901**	*HSP90AA1*	**Heat shock protein HSP 90-alpha**	2	2	3.68	100	40.7	1.24E-04	4
**8**	**Q9DBJ1**	*PGAM1*	**Phosphoglycerate mutase 1**	1	1	5.51	99.4	38.7	1.07E-04	1
**9**	**P60710**	*ACTB*	**Actin, cytoplasmic 1**	3	3	10.4	100	29.8	1.09E-04	2
10	**O08599**	*STXBP1*	Syntaxin-binding protein 1	2	2	2.86	100	75.5	1.06E-04	5
**11**	**P61982**	*YWHAG*	**14-3-3 protein gamma**	2	1	11.74	99.73	38.8	1.06E-04	3
**12**	**P10126**	*EEF1A1*	**Elongation factor 1-alpha 1**	1	1	6.28	100	22.4	1.10E-04	5
13	P17742	*PPIA*	Peptidyl-prolyl cis-trans isomerase A	1	1	5.49	99.77	209.9	8.06E-05	5
14	**O08553**	*DPYSL2*	Dihydropyrimidinase-related protein 2	2	2	2.8	99.73	105	2.05E-04	5
15	Q91ZJ5	*UGP2*	UTP--glucose-1-phosphate uridylyltransferase	2	2	7.48	99.4	100.3	1.01E-04	1

#	Accession number	Gene	Recommended protein name (UniProt)	Peptides	Unique peptides	Sequence coverage, %	Confidence, % *	emPAI †	Adjusted p-value‡	Protein function
**16**	**O35945**	*ALDH1A7*	**Aldehyde dehydrogenase, cytosolic 1**	1	1	2.59	99.45	93.5	1.01E-04	1
17	**P05063**	*ALDOC*	**Fructose-bisphosphate aldolase C**	3	3	12.67	100	77.4	7.31E-05	1
18	P68368	*TUBA4A*	Tubulin alpha-4A chain	7	1	21.88	99.73	59.3	8.95E-05	2
19	P80314	*CCT2*	T-complex protein 1 subunit beta	1	1	2.8	100	55.9	1.87E-04	4
**20**	**Q8C196**	*CPS1*	Carbamoyl-phosphate synthase [ammonia], mitochondrial	2	2	2.47	98.78	45.8	1.18E-04	1
21	**Q04447**	*CKB*	**Creatine kinase B-type**	5	5	13.91	95.52	41.7	1.05E-04	1
22	Q63810	*PPP3R1*	Calcineurin subunit B type 1	1	1	10	100	40.6	8.68E-05	3
**23**	Q9D6F9	*TUBB4A*	Tubulin beta-4A chain	1	1	4.05	99.77	30.7	1.71E-04	2
**24**	A1L317	*KRT24*	Keratin, type I cytoskeletal 24	1	1	3.13	99.4	28.9	8.68E-05	2
**25**	Q3THS6	*MAT2A*	S-adenosylmethionine synthase isoform type-2	1	1	5.06	100	24.8	1.06E-04	1
26	**Q78PY7**	*SND1*	**Staphylococcal nuclease domain-containing protein 1**	1	1	2.2	100	19.3	7.58E-05	5
27	P62814	*ATP6V1B2*	V-type proton ATPase subunit B, brain isoform	1	1	4.11	99.4	18.9	7.78E-05	2
**28**	P03995	*GFAP*	Glial fibrillary acidic protein	1	1	2.56	100	17.9	7.31E-05	2
**29**	P55066	*NCAN*	Neurocan core protein	1	1	1.66	100	16.7	9.66E-05	5

*The confidence is a relative value defining the strength of the protein detection (detectable power) and considering peptides with scores above the homology and the identity threshold score at certain searching setting, e.i., mass tolerance (at MS and MS/MS levels), number of allowed missing sites for the digesting enzyme, correlation between possible and confident sequence coverage with molecular mass of the detected protein, fragments coverage of peptide, etc.

† emPAI—is an Exponentially Modified Protein Abundance index used typically to approximate relative abundance (quantitation) of the proteins in a mixture based on protein coverage by the peptide matches in a database search result.

‡ Raw p-value (*p*-value cut-off of 0.025) was adjusted using a Bonferroni correction.

Here, and in the subsequent table, moonlighting proteins are shown in bold. Numbers in the Functional group column designate the following protein functions: 1. metabolic enzymes; 2. proteins involved in cytoskeleton formation and trafficking; 3. proteins involved in signal transduction and enzyme activity regulation; 4. protective proteins and components of the ubiquitin–proteasome system; 5. protein regulators of gene expression, cell division, and differentiation.

Supplementary Table S2. Individual mouse brain proteins bound exclusively to the PK peptide immobilized on CNBr-Sepharose

#	Accession number	Gene	Protein name (Uniprot)	Peptides	Unique peptides	Sequence coverage %	Confidence, %	emPAI	Adjusted p-value	Functional group
1	E9Q557	*DESP*	Desmoplakin	15	15	6	99.73	45.82	2.71E-04	2
2	O35887	*CALU*	Calumenin	4	4	12.7	100	44.14	1.07E-04	2
3	O70456	*1433S*	14-3-3 protein sigma	8	6	24.6	100	32.85	4.99E-04	3
4	O88569	*ROA2*	Heterogeneous nuclear ribonucleoproteins A2/B1	3	3	11.6	100	102.55	4.65E-04	5
**5**	P10107	*ANXA1*	Annexin A1	2	2	5.5	100	104.97	2.11E-04	3
6	P07356	*ANXA2*	Annexin A2	8	8	26.3	96.49	47.73	1.17E-04	3
7	P08113	*ENPL*	Endoplasmin	3	3	4.1	97.56	82.29	2.54E-04	2
8	P08228	*SODC*	Superoxide dismutase [Cu-Zn]	3	3	23.4	95.52	42.7	2.54E-04	4
**10**	**P09103**	***PDIA1***	**Protein disulfide-isomerase**	2	2	4.9	99.4	59.28	1.16E-04	4
**10**	**P17751**	***TPIS***	**Triosephosphate isomerase**	2	2	7.7	99.77	55.92	1.87E-04	1
11	**P17183**	***ENOG***	**Gamma-enolase**	3	1	9.4	99.77	104.97	7.31E-05	1
**12**	P20029	*BIP*	Endoplasmic reticulum chaperone BiP	11	10	21.5	96.67	40.65	7.31E-05	4
**13**	**P101**	***EF1A1***	**Elongation factor 1-alpha 1**	5	5	10.6	100	46.5	5.55E-05	5
14	P14206	*RSSA*	40S ribosomal protein SA	2	2	8.5	100	45.82	7.27E-05	5
**15**	**P14211**	***CALR***	**Calreticulin**	4	4	23.8	100	38.79	8.64E-05	3
16	P16627	*HS71L*	Heat shock 70 kDa protein 1-like	4	1	10	100	28.03	1.11E-04	4
**17**	P17156	*HSP72*	Heat shock-related 70 kDa protein 2	5	1	11.2	99.73	27.54	9.73E-05	4
18	**P20152**	***VIME***	**Vimentin**	12	12	22.7	99.73	21.76	1.66E-04	2
**19**	P21107	*TPM3*	Tropomyosin alpha-3 chain	8	2	18.9	99.73	18.65	1.05E-04	2
**20**	P26039	*TLN1*	Talin-1	2	2	1	99.73	18.14	1.41E-04	2
**21**	**P29595**	***NEDD8***	**NEDD8**	2	2	17.3	97.4	100.27	1.09E-04	5
22	P37804	*TAGL*	Transgelin	3	3	10.4	97.4	26.57	1.53E-04	2
**23**	P45591	*COF2*	Cofilin-2	2	1	16.9	100	26.57	2.55E-04	3
24	P48036	*ANXA5*	Annexin A5	2	2	9.4	100	25.96	1.86E-04	3
25	P48428	*TBCA*	Tubulin-specific chaperone A	2	2	11.1	96.49	40.57	1.86E-04	4
**26**	P48678	*LMNA*	Prelamin-A/C	11	11	19.2	98.18	39.29	1.55E-04	5
27	P49312	*ROA1*	Heterogeneous nuclear ribonucleoprotein A1	3	3	12.8	98.18	30.68	1.01E-04	5
28	P38647	*GRP75*	Stress-70 protein, mitochondrial	3	3	4.9	95.23	30.68	1.06E-04	4
29	P42208	*02-Sep*	Septin-2	2	2	7.2	95.76	20.46	1.06E-04	3
30	P43274	*H14*	Histone H1.4	2	2	12.3	97.28	209.85	7.61E-05	5
31	P52503	*NDUS6*	NADH	2	2	21.6	96.05	72.39	1.98E-04	1

#	Accession number	Gene	Protein name (Uniprot)	Peptides	Unique peptides	Sequence coverage %	Confidence, %	emPAI	Adjusted p-value	Functional group
			dehydrogenase [ubiquinone] iron-sulfur protein 6, mitochondrial							
32	P56480	*ATPB*	ATP synthase subunit beta, mitochondrial	4	4	15.3	100	71.96	8.25E-05	1
33	**P62259**	***1433E***	**14-3-3 protein epsilon**	3	1	12.2	97.67	65.12	8.25E-05	3
34	P58252	*EF2*	Elongation factor 2	2	2	3.4	99.73	59.84	1.06E-04	5
35	P58771	*TPM1*	Tropomyosin alpha-1 chain	10	2	23.9	100	40.65	1.06E-04	2
**36**	P60843	*IF4A1*	Eukaryotic initiation factor 4A-I	4	4	9.6	99.45	31.28	1.09E-04	5
37	P62806	*H4*	Histone H4	4	4	40.8	99.45	25.41	1.09E-04	5
38	P62918	*RL8*	60S ribosomal protein L8	2	2	10.5	98.38	93.55	6.97E-05	5
39	P62960	*YBOX1*	Y-box-binding protein 1	6	6	30.1	100	93.55	1.06E-04	5
40	P62984	*RL40*	Ubiquitin-60S ribosomal protein L40	4	4	36.7	100	31.88	7.58E-05	5
41	**P63260**	***ACTG***	**Actin, cytoplasmic 2**	13	1	42.4	100	29.79	9.67E-05	2
42	P68134	*ACTS*	Actin, alpha skeletal muscle	9	3	26.6	100	28.88	1.13E-04	2
43	P68040	*RACK1*	Receptor of activated protein C kinase 1	3	3	11	95.51	22.36	8.95E-05	3
44	P68254	*1433T*	14-3-3 protein theta	3	1	13.1	100	22.12	8.95E-05	3
45	P68373	*TBA1C*	Tubulin alpha-1C chain	5	5	18.7	99.77	24.83	1.24E-04	2
46	P99024	*TBB5*	Tubulin beta-5 chain	4	2	13.3	99.73	167.52	1.24E-04	2
47	P84078	*ARF1*	ADP-ribosylation factor 1	2	2	13.3	99.45	165.42	3.20E-04	3
48	P84244	*H33*	Histone H3.3	2	2	11.8	99.45	157.29	1.56E-04	5
**49**	Q02257	*PLAK*	Junction plakoglobin	4	4	6.4	99.77	135.3	1.56E-04	2
**50**	Q05186	*RCN1*	Reticulocalbin-1	2	2	12.9	100	110.94	5.23E-05	3
**51**	Q3THE2	*ML12B*	Myosin regulatory light chain 12B	3	1	23.3	99.77	77.36	1.09E-04	2
52	Q60817	*NACA*	Nascent polypeptide-associated complex subunit alpha	2	2	13	98.7	77.36	2.71E-05	5
**53**	Q61696	*HS71A*	Heat shock 70 kDa protein 1A	5	1	12.3	98.7	75.46	5.59E-05	4
54	Q62048	*PEA15*	Astrocytic phosphoprotein PEA-15	2	2	27.7	99.4	75.46	5.59E-05	3
55	Q64727	*VINC*	Vinculin	2	2	3.8	99.4	51.32	3.38E-05	2

#	Accession number	Gene	Protein name (Uniprot)	Peptides	Unique peptides	Sequence coverage %	Confidence, %	emPAI	Adjusted p-value	Functional group
**56**	**Q68FD5**	***CLH1***	**Clathrin heavy chain 1**	5	5	5.3	99.4	49.51	4.69E-05	2
57	Q6IRU2	*TPM4*	Tropomyosin alpha-4 chain	5	4	12.9	100	41.68	8.68E-05	2
58	Q7TPR4	*ACTN1*	Alpha-actinin-1	8	5	13.7	99.73	40.57	6.17E-05	2
59	Q8BFR5	*EFTU*	Elongation factor Tu, mitochondrial	2	2	2.7	100	38.72	4.46E-05	5
60	Q8BG05	*ROA3*	Heterogeneous nuclear ribonucleoprotein A3	4	4	15	100	34.03	5.09E-05	5
61	Q8BKC5	*IPO5*	Importin-5	2	2	4.1	99.82	33.36	6.68E-05	2
**62**	**Q8BTM8**	***FLNA***	**Filamin-A**	18	17	10.3	100	32.85	7.48E-05	2
63	Q8C1B7	*11-Sep*	Septin-11	3	3	13	100	31.88	4.12E-05	3
64	Q8CGP6	*H2A1H*	Histone H2A type 1-H H2A	2	2	12.5	99.77	28.88	4.86E-05	5
65	Q8VDD5	*MYH9*	Myosin-9	20	20	11.7	99.77	25.65	4.71E-05	2
66	Q8VHX6	*FLNC*	Filamin-C	5	4	2.9	100	24.96	1.27E-04	2
67	Q922R8	*PDIA6*	Protein disulfide-isomerase A6	2	2	6.4	98.7	24.83	1.17E-04	4
68	Q99K48	*NONO*	Non-POU domain-containing octamer-binding protein	2	2	6.6	100	22.44	2.72E-05	5
69	Q99PT1	*GDIR1*	Rho GDP-dissociation inhibitor 1	2	2	8.3	99.45	22.36	2.64E-05	3
70	Q9CQ19	*MYL9*	Myosin regulatory light polypeptide 9	3	1	23.3	100	21.9	3.04E-05	2
71	Q9CQV8	*1433B*	14-3-3 protein beta/alpha	3	1	13	100	20.74	2.10E-05	3
72	Q9JHU4	*DYHC1*	Cytoplasmic dynein 1 heavy chain 1	2	2	0.6	100	20.4	2.44E-05	2
73	Q9JKF1	*IQGA1*	Ras GTPase-activating-like protein IQGAP1	2	2	2.1	100	19.29	2.65E-05	3
74	Q9Z2U0	*PSA7*	Proteasome subunit alpha type-7	3	3	12.9	100	19.29	5.88E-05	4
75	Q9QXS1	*PLEC*	Plectin	2	2	0.6	100	18.92	5.88E-05	2
76	Q9QXS6	*DREB*	Drebrin	5	5	9.1	98.7	18.92	1.59E-04	2
77	Q9Z1Q5	*CLIC1*	Chloride intracellular channel protein 1	2	2	10	98.7	18.65	9.19E-06	2
